# Regulatory effects of *Trichinella spiralis* and a serine protease inhibitor on the endoplasmic reticulum stress response of intestinal epithelial cells

**DOI:** 10.1186/s13567-022-01036-x

**Published:** 2022-03-03

**Authors:** Jingyun Xu, Zixuan Pang, Jinpeng Zhang, Shuang Xia, Ruibiao Wang, Yuheng Zhang, Jingbo Zhen, Xuewei Song, Lihao Lin, Feng Sun, Xinxin Xuan, Yixin Lu

**Affiliations:** 1 Heilongjiang Provincial Key Laboratory of Zoonosis, College of Veterinary Medicine, Northeast Agricultural University, 600 Changjiang Street, Harbin, 150030 China; 2 College of Veterinary Medicine, Sichuan Agricultural University, 211 Huimin Street, Chengdu, 611130 China

**Keywords:** Endoplasmic reticulum stress response, apoptosis, *Trichinella spiralis*, TsKaSPI

## Abstract

The accumulation of unfolded or misfolded proteins in the endoplasmic reticulum can cause an endoplasmic reticulum stress (ERS) response. If ERS continues or cannot be alleviated, it will cause the production of proapoptotic factors and eventually lead to apoptosis. Therefore, this study mainly explored whether *Trichinella spiralis* Kazal-type serine protease inhibitor (TsKaSPI) contributed to the invasion of intestinal epithelial cells during the infectious stage of *T. spiralis* by regulating ERS. First, in the *T. spiralis* infection model, H&E staining was used to analyse the damage to jejunum tissue, a TUNEL assay was used to examine cell apoptosis, and the expression of ERS-related and apoptosis-related molecules was also measured. The results showed that ERS occurred during the intestinal phase of *T. spiralis* infection, while remission began during the cyclic phase. Then, we selected TsKaSPI, one of the important components of *T. spiralis* ES antigens, for in vitro experiments. The results showed that TsKaSPI could induce apoptosis in a porcine small intestinal epithelial cell line (IPEC cells) by activating ERS and promote activation of the NF-κB signalling pathway. Inhibition experiments confirmed that the occurrence of ERS was accompanied by the activation of NF-κB, and the two processes regulated each other. Finally, we conducted in vivo experiments and administered TsKaSPI to mice. The results confirmed that TsKaSPI could activate ERS and lead to apoptosis in intestinal epithelial cells. In conclusion, *T. spiralis* infection and TsKaSPI can promote cell apoptosis by activating the ERS response in intestinal epithelial cells and activate the NF-κB signalling pathway to promote the occurrence and development of inflammation.

## Introduction

Trichinellosis is a serious foodborne zoonotic parasitic disease with a worldwide distribution [1, 2]. Intestinal invasion is an important stage of *Trichinella spiralis* (*T. spiralis*) infection, which determines the occurrence and development of the disease [3]. *T. spiralis* excretion-secretion (ES) antigens may play key roles in the pathogenic process [4]. Serine protease inhibitor (SPI), which is an important component of ES antigens, can not only regulate enzyme activity to interfere with protein metabolism but also participate in some important physiological and pathological processes, such as complement activation, the inflammatory response and apoptosis [5–7]. Piekarska et al. found that the intestinal stage of *T. spiralis* infection could induce apoptosis in cells in the villous lamina propria [8, 9]. Therefore, we hypothesized that *T. spiralis* SPI (TsSPI) may be involved in intestinal epithelial cell apoptosis induced by *T. spiralis* infection, but the specific mechanism remains to be further explored.

Endoplasmic reticulum stress (ERS)-mediated apoptosis plays a key role in many diseases, such as infectious diseases, inflammatory diseases, neurodegenerative diseases, atherosclerosis, diabetes, and cancer [10–14]. In recent years, pathogens such as parasites, bacteria and viruses, which are important ER stressors, can initiate the ERS response [15–20]. Dias-Teixeira et al. [21] found that infection with *Leishmania amazonensis* could induce ERS in macrophages. Yu et al. [22] found that *Schistosoma japonicum* infection could significantly upregulate ERS markers in the livers of mice. Wang et al. [23] found that *Toxoplasma gondii* could induce neural stem cell apoptosis through ERS. Although studies of ERS have become a hot spot, there have been no relevant studies on the relationship between TsSPI and ERS in intestinal epithelial cells. Thus, the aim of this study was to analyse whether TsSPI regulated the host intestinal inflammatory response by regulating ERS-mediated apoptosis signalling during *T. spiralis* invasion. The results of our work will provide insights into the pathogenesis of trichinosis.

## Materials and methods

### Animals and cells

Specific pathogen-free (SPF) 6–8-week-old female BALB/c mice (*n* = 100 in total) were purchased from Harbin Medical University. All animal husbandry and experimental procedures were performed in accordance with the Chinese Animal Management Ordinance and the animal experiment standards approved by the Animal Management Committee of Northeast Agricultural University. All mice had free access to food and water, and they were maintained under SPF conditions with 70 ± 10% humidity and a temperature of 20 ± 2 ℃.

Porcine small intestinal epithelial cells (IPEC-J2 cells) were donated by Harbin Veterinary Research Institute.

### Parasite and protein preparation

*Trichinella spiralis* (strain ISS533) was cultured in KM mice, and muscle larvae (ML) were isolated from the muscles of infected KM mice as previously described [24]. The excretory-secretory products of *T. spiralis* ML (MES) were prepared and collected as previously described [25–27]. *T. spiralis* ML were freshly collected from mouse muscle on Day 40 post-infection, washed three times with physiological saline, and then cultured in RPMI-1640 medium (Meilunbio, China) supplemented with 10% foetal bovine serum (FBS), 100 U/mL penicillin and 100 U/mL streptomycin (Solarbio, China) at 37 ℃, 5% CO_2_ for 48 h. The culture supernatant containing MES was concentrated by centrifugation, and then PEG2000 was used for concentration before the solution was filtered through a 0.22-micron syringe filter (Merck, Germany).

The pET-30a-TsKaSPI positive expression bacterial solution was prepared and stored at −80 ℃ [28]. Then, purification was performed by a nickel column and renaturation. The protein concentrations of the prepared MES and pET-30a-TsKaSPI were determined by bicinchoninic acid (BCA) assays (Takarabio, China). Endotoxin contamination was removed by a ToxOut™ High Capacity Endotoxin Removal Kit (GenScriptbio, China).

### Experimental infection

To analyse the effect of *T. spiralis* on ERS in the jejunum, each BALB/c mouse was orally infected with 200 infective *T. spiralis* ML on Day 0. On Days 3, 7, and 15 post-infection, six mice were randomly selected and sacrificed. The jejunum was collected, and Day 0 was designated the uninfected control. Time points were selected according to the developmental stage of the worm in the host. Day 3 and Day 7 were the intestinal phase of *T. spiralis*; Day 15 was the stage at which newborn larvae (NBL) migrated in the circulatory system and tissue of the host.

### Histologic assessment

For histological analysis, a 2 cm long segment of jejunum was excised from each mouse, flushed gently with cold phosphate buffered saline (PBS), fixed with 10% neutral formalin overnight, embedded in paraffin, cut into 5 μm sections and stained with haematoxylin and eosin (H&E) according to standard procedures. Intestinal pathology was evaluated by a single blinded scorer using a validated scoring system [29, 30] (Table 1). Villus length and crypt depth in the jejunum were measured using Image-Pro Plus software.

**Table 1:** **Histopathological scoring**

Indicators	Score			
	0	1	2	3
Epithelial integrity	No change	Shedding of < 10 epithelial cells per lesion	Shedding of 11–20 epithelial cells per lesion	Epithelial ulceration
Central lacteal expansion	No change	Mild	Moderate	Profound
leukocyte infiltration	< 10 leukocytes per field	11–15 leukocytes per field	16–20 leukocytes per field	> 20 leukocytes per field
Submucosal oedema	No change	Mild	Moderate	Profound
Mucosal hyperaemia	None	Mild	Moderate	Severe
Lymphoid necrosis of Peyer’s patches	None	Mild	Moderate	Severe

### TUNEL staining

Intestinal epithelial cell apoptosis in the jejunum was identified by TUNEL staining according to the manufacturer’s instructions [14]. Apoptotic cells (green staining) were observed.

### Quantitative real-time PCR

The relative gene expression of PERK, IRE1, ATF6, and Bip in jejunum tissues was evaluated by quantitative real-time PCR (qPCR). Table 2 shows the primer sequences used to detect each gene. Total RNA was extracted from jejunum tissues using a total RNA extraction kit (Solarbio, China). After cDNA was synthesized from total RNA using the Prime Script 1st Strand cDNA Synthesis Kit (Takara, Japan), qPCR was performed using a Roche Light Cycler 480 system. The results were calculated using the 2^−∆∆Ct^ method [31].

**Table 2:** **Primers of the detected genes**

	Primers	Sequence	Access number	Source
β-Actin	Forward	5′-GTGACGTTGACATCCGTAAAGA-3′	NM_007393	*Mus musculus*
	Reverse	5′-GCCGGACTCATCGTACTCC-3′		
IRE1	Forward	5′-TAAGGACAACCCTACCTACAC-3′	NM_023913	*Mus musculus*
	Reverse	5′-AATTCACGAGCAATGACG-3′		
PERK	Forward	5′-GCACTTTAGATGGACGAATCGC-3′	NM_010121	*Mus musculus*
	Reverse	5′-GCTGTCGCCATATAAGGAAAGG-3′		
ATF6	Forward	5′-CGGTCCACAGACTCGTGTTC-3′	NM_001081304	*Mus musculus*
	Reverse	5′-GCTGTCGCCATATAAGGAAAGG-3′		
Bip	Forward	5′-GCATCACGCCGTCGTATGT-3′	NM_001163434	*Mus musculus*
	Reverse	5′-ATTCCAAGTACATCCGATGAG-3′		
β-Actin	Forward	5′-GACTACCTCCTGTCTGCTGAG-3′	XM_021086047	*Sus scrofa* (pig)
	Reverse	5′-GGTTTCTGTGCCTCACTCCC-3′		
IRE1	Forward	5′-GCCTCCTGTTAGTGTCCACC-3′	XM_021086462	*Sus scrofa* (pig)
	Reverse	5′-ACACTGGCCCTTGGATGATG-3′		
PERK	Forward	5′-GACTGTGACTTGGAGGACGG-3′	XM_003124925	*Sus scrofa* (pig)
	Reverse	5′-AGGCAGTCGGTTCATTCTGG-3′		
ATF6	Forward	5′-ACAGAAACCACTAGTATCAGCAGG-3′	XM_021089515	*Sus scrofa* (pig)
	Reverse	5′-CCTTCTGCGGATGGCTTCAA-3′		
Bip	Forward	5′-ACCACCTACTCGTGCGTTG-3′	XM_001927795	*Sus scrofa* (pig)
	Reverse	5′-CGTCGAAGACCGTGTTCTCA-3′		
TLR2	Forward	5′-GGTGTGCTGCAAGGTCAAC-3′	NM_213761	*Sus scrofa* (pig)
	Reverse	5′-GAGAAGAAGCCTGATGGGGG-3′		
TLR4	Forward	5′-TGCTTTCTCCGGGTCACTTC-3′	NM_001113039	*Sus scrofa* (pig)
	Reverse	5′-TTAGGAACCACCTGCACGC-3′		
Bcl-2	Forward	5′-GGATAACGGAGGCTGGGATG-3′	XM_021099593	*Sus scrofa* (pig)
	Reverse	5′-TTATGGCCCAGATAGGCACC-3′		
Bcl-xL	Forward	5′-GGTCGCATTGTGGCCTTTTT-3′	NM_214285	*Sus scrofa* (pig)
	Reverse	5′-TCCACAAAAGTGTCCCAGCC-3′		
Bax	Forward	5′-GCCCTTTTGCTTCAGGGTTTC-3′	XM_003127290	*Sus scrofa* (pig)
	Reverse	5′-CAATGCGCTTGAGACACTCG-3′		
Fas	Forward	5′-GGGTTCTCCTGTCACTGGTAT-3′	NM_213839	*Sus scrofa* (pig)
	Reverse	5′-CAGCATGTTTCCGTTTGCCA-3′		

### Western blotting

The jejunum tissues of each group were cut into small pieces and weighed. RIPA lysis buffer and 1% PMSF (Solarbio, China) were added at a ratio of 150–250 μL of lysis buffer per 20 mg of jejunum tissue and then ultrasonically lysed. After lysis, the samples were centrifuged at 10 000–14 000 × *g* for 3–5 min to obtain the supernatant. The protein concentration was determined using a BCA assay kit (Takarabio, China). A total of 50 µg of jejunum protein was boiled with 5× SDS-PAGE sample loading buffer (Biosharp, China) and separated using SDS-PAGE. The proteins were blotted onto a nitrocellulose filter membrane (Biosharp, China). The membrane was placed into blocking buffer (5% nonfat milk) for 2 h at room temperature. Subsequently, the membrane was incubated with anti-β-actin (1:1000), anti-PERK (1:1500), anti-IER1 (1:1500), anti-ATF6 (1:500), anti-Bip (1:1500), anti-p-JNK (1:1000), anti-JNK (1:1000), anti-CHOP (1:500), and anti-caspase-12 (1:500) (diluted in blocking buffer, Wanleibio, China) at 4 °C overnight. After being washed three times using PBS with Tween 20 (PBST), the membrane was incubated with peroxidase-conjugated secondary antibodies diluted in 5% nonfat milk (1:4000) on a shaker for 2 h at room temperature. After the membrane was washed three times, ultrasensitive ECL chemiluminescence reagent (Meilunbio, China) was added to the membrane, and the membrane was exposed. The bands were quantified using densitometry and analysed with ImageJ.

### Cell culture

Single-cell suspensions of IPECs were prepared. Subsequently, 1 × 10^6^/well IPECs in 1 mL of RPMI-1640 containing 10% FBS and 100 U/mL penicillin/streptomycin (Meilunbio, China) were cultured in 6-well plates at 37 ℃ at 5% CO_2_ for 24 h in the presence of PBS, tunicamycin (Tm, ERS activator) (Meilunbio, China), TsES and TsKaSPI. The effects of Tm, TsES, and TsKaSPI on cell proliferation were evaluated by a cell counting kit 8 (CCK8) assay using a spectrophotometer, and the optimal reaction concentrations of Tm, TsES, TsKaSPI and IPECs were further determined. Culture supernatants were used to examine IL-1β, IL-6, and TNF-α using corresponding ELISA kits (ProteLightbio, China) according to the manufacturer’s instructions. Moreover, the relative gene expression of the PERK, IRE1, ATF6, Bip, TLR2, TLR4, Fas, Bax, Bcl-xL, and Bcl-2 in IPECs was evaluated by qPCR, and the primers for the target genes are shown in Table 2. The protein expression of PERK, IERK1, ATF6, Bip, p-JNK, JNK, CHOP, caspase-12, NF-κB p65, and p-NF-κB p65 in IPECs was determined by Western blotting. Furthermore, an Annexin V-FITC/PI cell apoptosis kit (Meilunbio, China) was used to examine apoptotic cells by FCM. First, 1 × 10^6^/mL IPECs were incubated with PBS, Tm, TsES, and TsKaSPI individually for 24 h, and then the cells were stained with PI and FITC to examine apoptotic cells.

### Inhibition experiment

To further analyse the key role of TsKaSPI in the mutual regulation of ERS and the inflammatory response, we conducted ERS inhibition experiments and NF-κB inhibition experiments separately. First, we divided the cells into the following groups: the IPEC + PBS group, IPEC + 4-PBA group, IPEC + TsKaSPI group, and IPEC + 4-PBA + TsKaSPI group. 4-PBA (Meilunbio, China) is a selective inhibitor of ERS. In the IPEC + 4-PBA + TsKaSPI group, we cultured 1 × 10^6^/mL IPECs with 50 μM 4-PBA for 1 h before adding TsKaSPI. Then, the protein expression of NF-κB p65 and p-NF-κB p65 in IPECs was determined by Western blotting. Culture supernatants were used to examine different cytokines using corresponding ELISA kits according to the manufacturer’s instructions. Second, we divided the cells into the following groups: the IPEC + PBS group, IPEC + PDTC group, IPEC + TsKaSPI group, and IPEC + PDTC + TsKaSPI group. PDTC (Meilunbio, China) is a specific inhibitor of NF-κB activation. In the IPEC + PDTC + TsKaSPI group, we cultured 1 × 10^6^/mL IPECs with 10 μM PDTC for 1 h before adding TsKaSPI. Then, the protein expression of Bip in IPECs was determined by Western blotting.

### TsKaSPI-induced ERS responses in mice

Thirty female BALB/c mice were randomly divided into 5 groups: the control group, PBS + adjuvant group, TsKaSPI primary immunization group, TsKaSPI secondary immunization group, and TsKaSPI third immunization group. The mice were intraperitoneally injected with 50 μg of TsKaSPI each time. Then, the mice were sacrificed 3 days after each immunization, and jejunum tissues were collected. The relative gene expression of PERK, IRE1, ATF6, and Bip in jejunum tissues was evaluated by qPCR, and the primers for the target genes are shown in Table 2. The protein expression of PERK, IERK1, ATF6, Bip, p-JNK, JNK, CHOP, and caspase-12 in jejunum tissues was determined by Western blotting as described above.

### Statistical analysis

All data are expressed as the mean ± SD. Statistical analysis was performed using GraphPad Prism 5. ImageJ software was used to quantify the band intensities. Differences between groups were assessed by one-way analysis of variance (ANOVA) with SPSS 11.5 software. *P* < 0.05 was considered statistically significant.

## Results

### Effects of *T. spiralis* infection on intestinal histology

**Figure 1:**
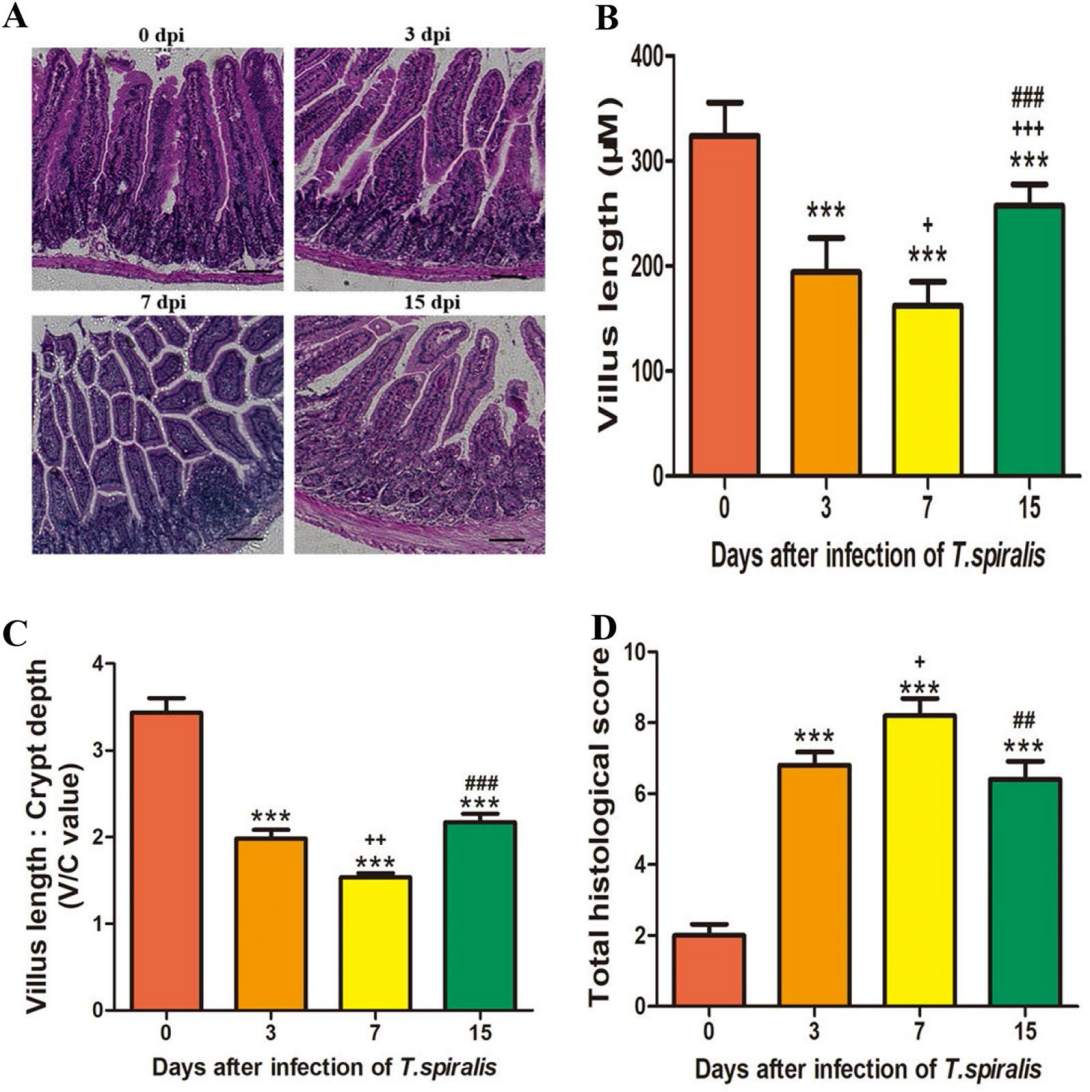
**H&E staining to evaluate pathological damage in intestinal tissue.** Histopathological sections of the jejunum were collected from mice on Days 3, 7, and 15 post-infection (dpi) (**A**). Villus length (**B**) and crypt depth of the jejunum were measured using Image-Pro Plus software. The ratio of villus length/crypt depth is shown in **C**, and the intestinal pathology score is shown in **D**. The data are expressed as the mean ± SD. *P < 0.05, **P < 0.01, ***P < 0.001 compared with the 0 dpi group, ^+^*P* < 0.05, ^++^*P* < 0.01, ^+++^*P* < 0.001 compared with the 3 dpi group, ^#^*P* < 0.05, ^##^*P* < 0.01, ^###^*P* < 0.001 compared with the 7 dpi group.

The histological alterations in the jejunum after *T. spiralis* infection were consistent with the findings of other reports [32–34]. Analyses of histopathologic changes revealed that infection with *T. spiralis* caused villus loss. Moreover, inflammatory infiltration was observed in some regions of the jejunum, and the villus length/crypt depth ratio was also increased after infection with *T. spiralis*. These changes peaked on Day 7 post-infection (7 dpi). Histologic assessment of the jejunum revealed that infection with *T. spiralis* could induce intestinal inflammation, and inflammation was gradually relieved at 15 dpi (Figure 1).

### *Trichinella spiralis* infection induced intestinal epithelial cell apoptosis

**Figure 2:**
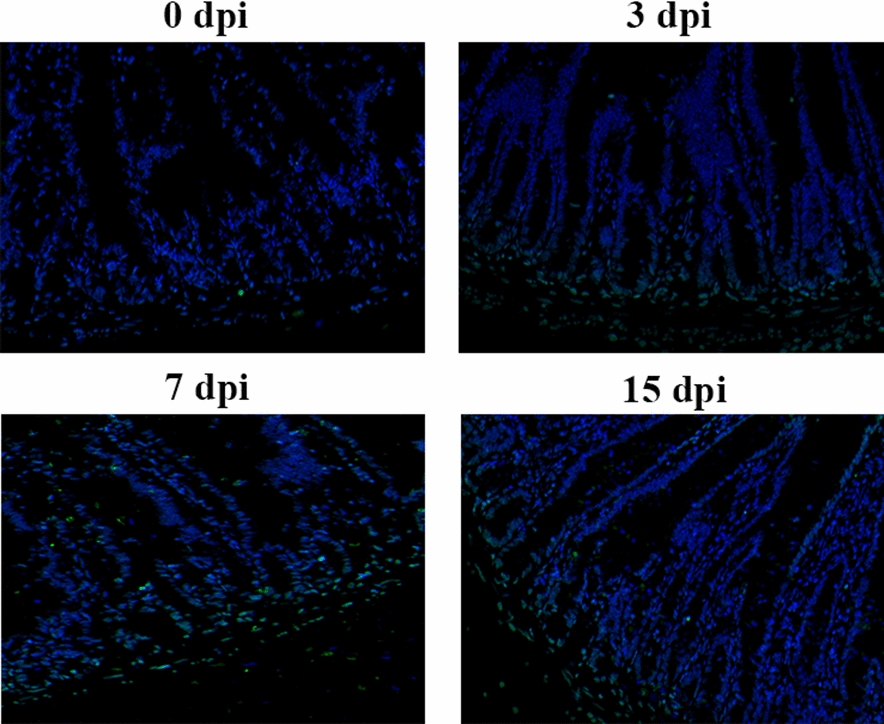
**TUNEL analysis of apoptosis in the intestines of mice infected with** ***T. spiralis*.** TUNEL staining in the jejunum of mice infected with *T. spiralis* at 0, 3, 7, and 15 dpi; apoptotic cells are stained green (original magnification ×200).

TUNEL staining revealed apoptosis in the intestines of mice infected with *T. spiralis*. Compared with that of uninfected mice, the jejunum showed an increased number of apoptotic cells at 3, 7, and 15 dpi, and the ratio of apoptotic cells was highest at 7 dpi (Figure 2).

### *Trichinella spiralis* infection induced ERS in the jejunum

**Figure 3:**
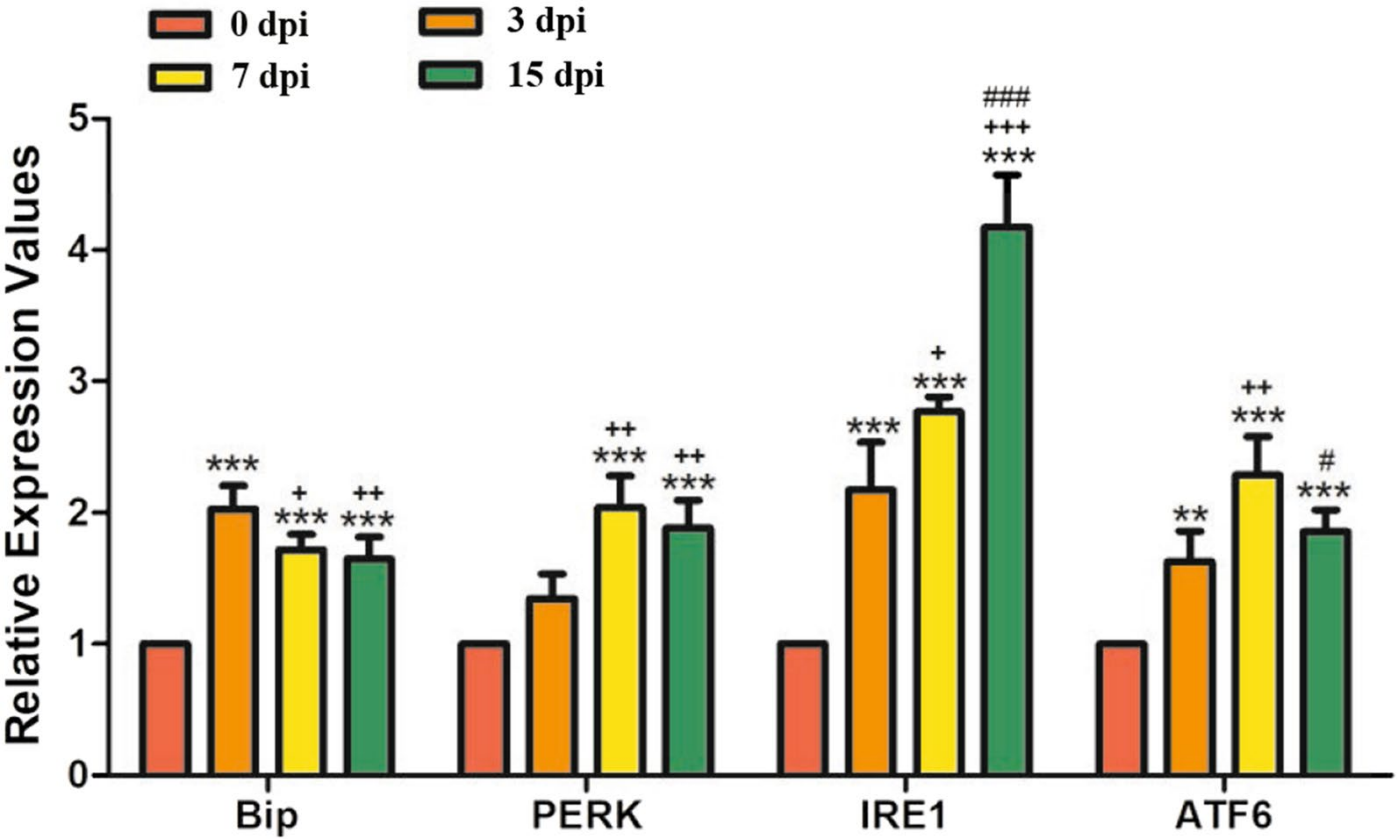
**qPCR analysis of the relative expression of ERS-related proteins in the jejunum.** Assays were performed in triplicate, and the data are expressed as the mean ± SD. *P < 0.05, **P < 0.01, ***P < 0.001 compared with the 0 dpi group, ^+^*P* < 0.05, ^++^*P* < 0.01, ^+++^*P* < 0.001 compared with the 3 dpi group, ^#^*P* < 0.05, ^##^*P* < 0.01, ^###^*P* < 0.001 compared with the 7 dpi group.

**Figure 4:**
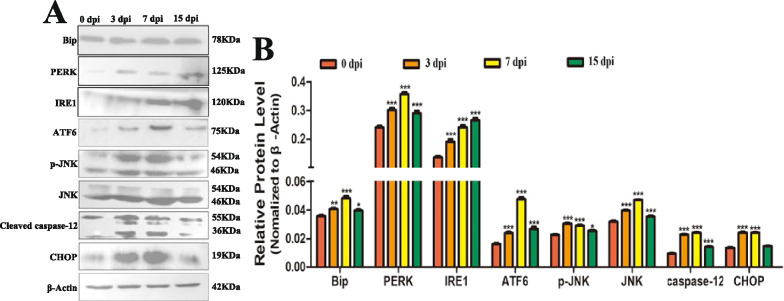
**Western blot analysis of ERS-related proteins and apoptosis-related proteins in the jejunum.** Assays were performed in triplicate, and the data are expressed as the mean ± SD. *P < 0.05, **P < 0.01, ***P < 0.001 compared with the 0 dpi group.

To analyse the effect of *T. spiralis* infection on the jejunum, the gene transcription levels of ATF6, Bip, IRE1, and PERK were measured by qPCR, and the protein expression levels of ATF6, Bip, IRE1, PERK, p-JNK, JNK, CHOP, and caspase-12 were measured by Western blotting. The results showed that the relative expression of ATF6, Bip, IRE1, and PERK was significantly increased during the early intestinal stage (3 dpi, 7 dpi) and newborn larvae (NBL) migration stage (15 dpi) compared to that of uninfected mice (Figure 3). The relative expression of ATF6, BIP, and PERK was highest at 7 dpi, while IRE1 expression was highest at 15 dpi compared with that in the other groups. ImageJ software was used to analyse the grey values of ATF6, Bip, IRE1, PERK, p-JNK, JNK, CHOP, and caspase-12 in each group. The results showed that the expression of ERS-related proteins and apoptosis-related proteins were significantly increased at 3, 7, and 15 dpi compared with those in the control group (Figure 4). Overall, the transcription and protein expression levels of ERS-related and apoptosis-related molecules were significantly increased during the early intestinal stage and NBL migration stage. Moreover, the expression levels gradually decreased during the NBL migration stage.

### TsKaSPI affected the proliferation of IPECs

**Figure 5:**
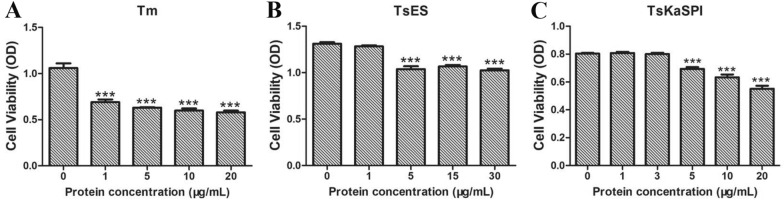
**Tm, TsES, and TsKaSPI affect IPEC proliferation.** Proliferation was measured by CCK-8 incorporation after the cells were stimulated with Tm (**A**), TsES (**B**), and TsKaSPI (**C**). The OD_450_ values were considered the cell proliferation index. The data from 3 independent experiments were analysed, and the data are expressed as the mean ± SD. *P < 0.05, **P < 0.01, ***P < 0.001 compared with the IPEC + PBS group, ^+^*P* < 0.05, ^++^*P* < 0.01, ^+++^*P* < 0.001 compared with the IPEC + Tm group, ^#^*P* < 0.05, ^##^*P* < 0.01, ^###^*P* < 0.001 compared with the IPEC + TsES group.

The effects of Tm, TsES, and TsKaSPI on cell proliferation were evaluated by a cell counting kit 8 (CCK8) assay using a spectrophotometer. The results showed that as a typical ERS inducer, a low dose of Tm could decrease the viability of IPECs (Figure 5A). There was no significant difference between the group treated with 1 µg/mL TsES and the control group (*P* > 0.05). The proliferation of IPECs incubated with 5 (*P* < 0.01), 15 (*P* < 0.001), and 30 µg/mL (*P* < 0.001) TsES was significantly suppressed compared with that of the control group (Figure 5B). When 1–3 µg/mL TsKaSPI was used to stimulate IPECs, the changes in cell proliferation were not significant. As the concentration of TsKaSPI increased to 5–20 µg/mL, the viability of IPECs significantly decreased (Figure 5C). Therefore, 1 µg/mL Tm, 1 µg/mL TsES, and 3 µg/mL TsKaSPI were selected as the working concentrations for subsequent experiments.

### TsKaSPI induced ERS in IPECs

**Figure 6:**
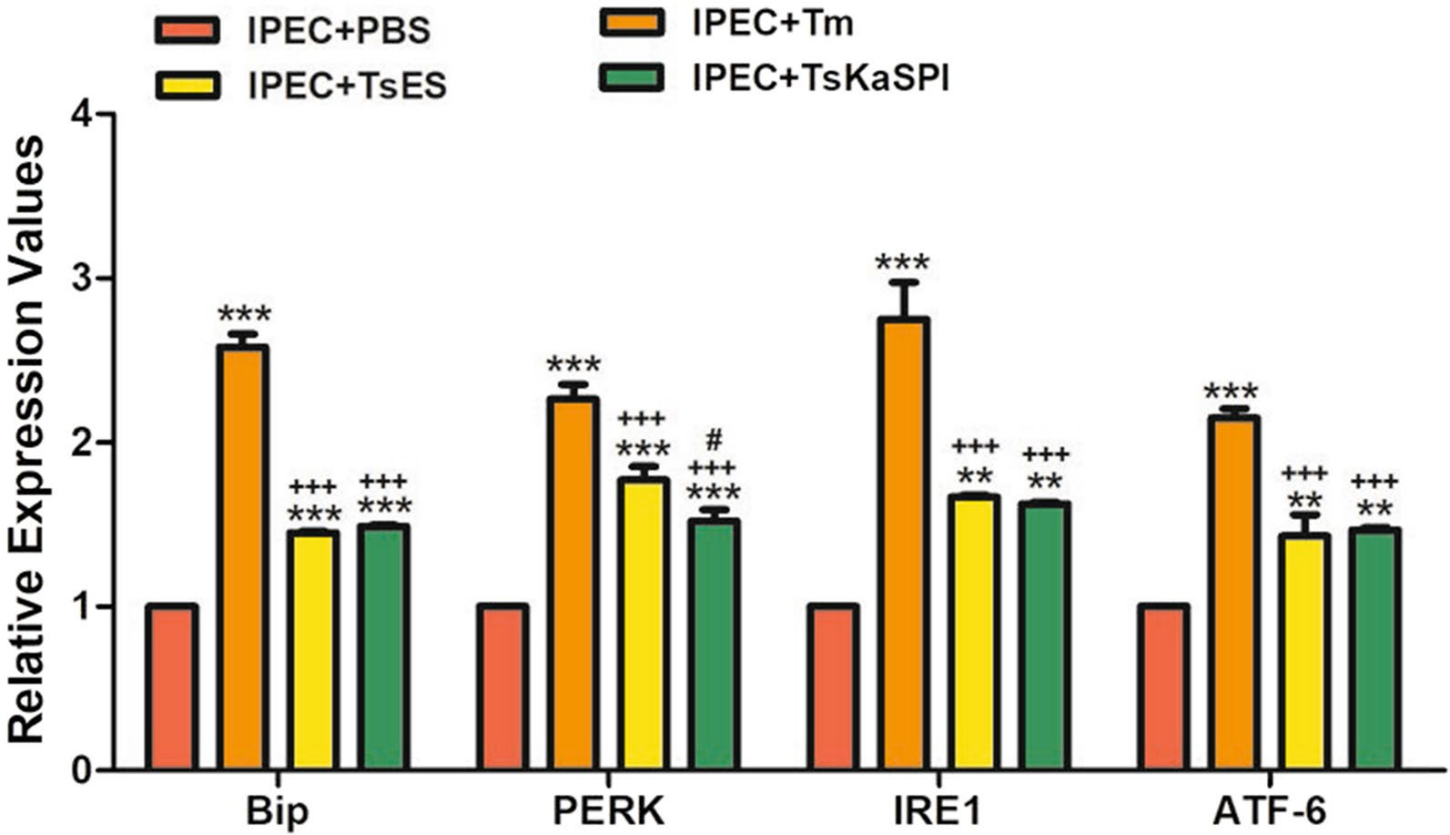
**qPCR analysis of the relative expression of ERS-related genes in IPECs treated with Tm, TsES, and TsKaSPI.** Assays were performed in triplicate, and the data are expressed as the mean ± SD. *P < 0.05, **P < 0.01, ***P < 0.001 compared with the IPEC + PBS group, ^+^*P* < 0.05, ^++^*P* < 0.01, ^+++^*P* < 0.001 compared with the IPEC + Tm group, ^#^*P* < 0.05, ^##^*P* < 0.01, ^###^*P* < 0.001 compared with the IPEC + TsES group.

qPCR was used to examine the gene transcription levels of ATF6, Bip, IRE1, and PERK in the control (IPEC + PBS) group, IPEC + Tm group, IPEC + TsES group and IPEC + TsKaSPI group. The results showed that the relative gene expression levels of ATF6, BIP, IRE1, and PERK were significantly increased in the IPEC + Tm, IPEC + TsES and IPEC + TsKaSPI groups compared with the control group (Figure 6).

**Figure 7:**
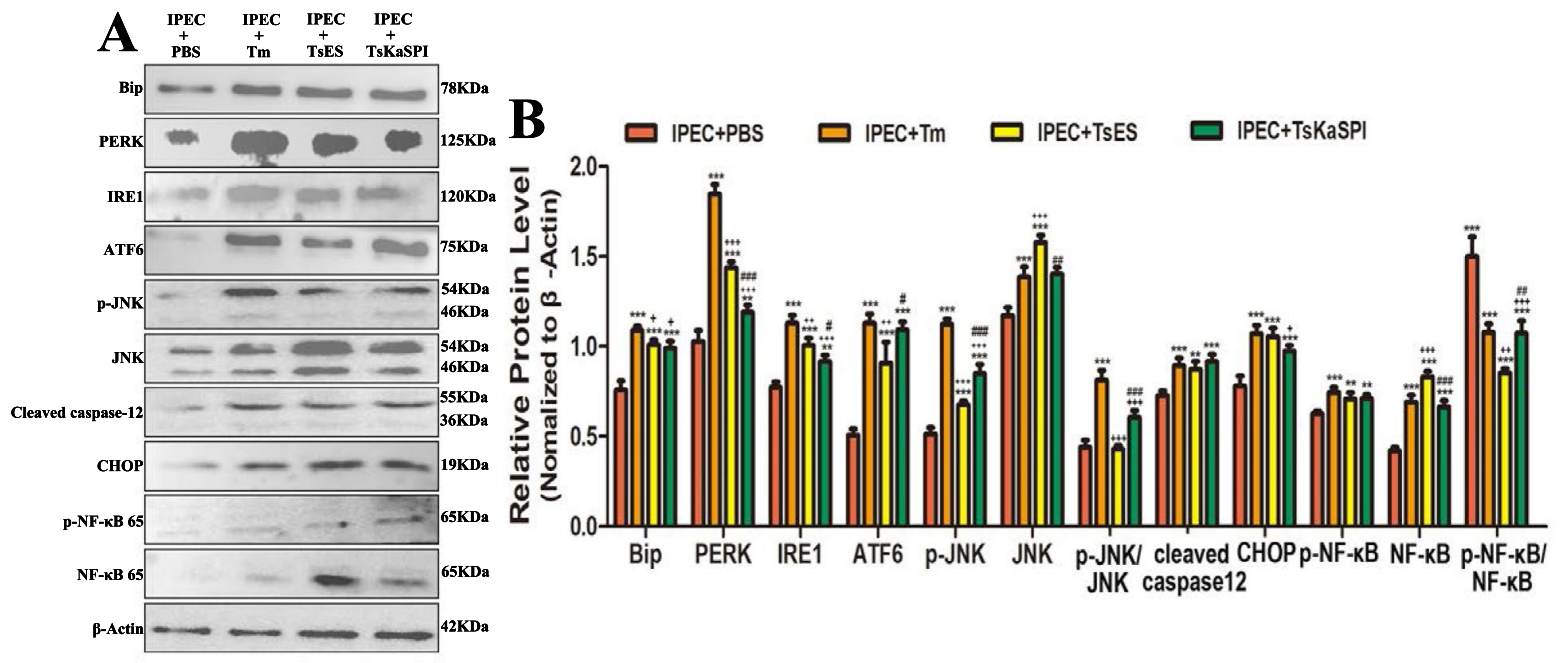
**Western blot analysis of the expression of ERS-related and apoptosis-related proteins and activation of the NF-κB signalling pathway in IPECs.** Assays were performed in triplicate, and the data are expressed as the mean ± SD. *P < 0.05, **P < 0.01, ***P < 0.001 compared with the IPEC + PBS group, ^+^*P* < 0.05, ^++^*P* < 0.01, ^+++^*P* < 0.001 compared with the IPEC + Tm group, ^#^*P* < 0.05, ^##^*P* < 0.01, ^###^*P* < 0.001 compared with the IPEC + TsES group.

Western blotting was used to examine the protein expression of ATF6, Bip, IRE1, PERK, p-JNK, JNK, CHOP, caspase-12, NF-κB p65, and p-NF-κB p65 in the control (IPEC + PBS), IPEC + Tm, IPEC + TsES and IPEC + TsKaSPI groups, and the grey values of the bands were analysed by ImageJ software (Figure 7). The expression levels of ATF6, Bip, IRE1, PERK, p-JNK, JNK, CHOP, cleaved caspase-12, NF-κB p65, and p-NF-κB p65 in the IPEC + Tm, IPEC + TsES and IPEC + TsKaSPI groups were significantly increased compared with those in the control group. The phosphorylation levels of JNK and NF-κB in the IPEC + Tm, IPEC + TsES and IPEC + TsKaSPI groups were also significantly higher than those in the control group. The results showed that both TsES and TsKaSPI could cause ERS in IPECs.

### TsKaSPI enhanced apoptosis in IPECs

**Figure 8:**
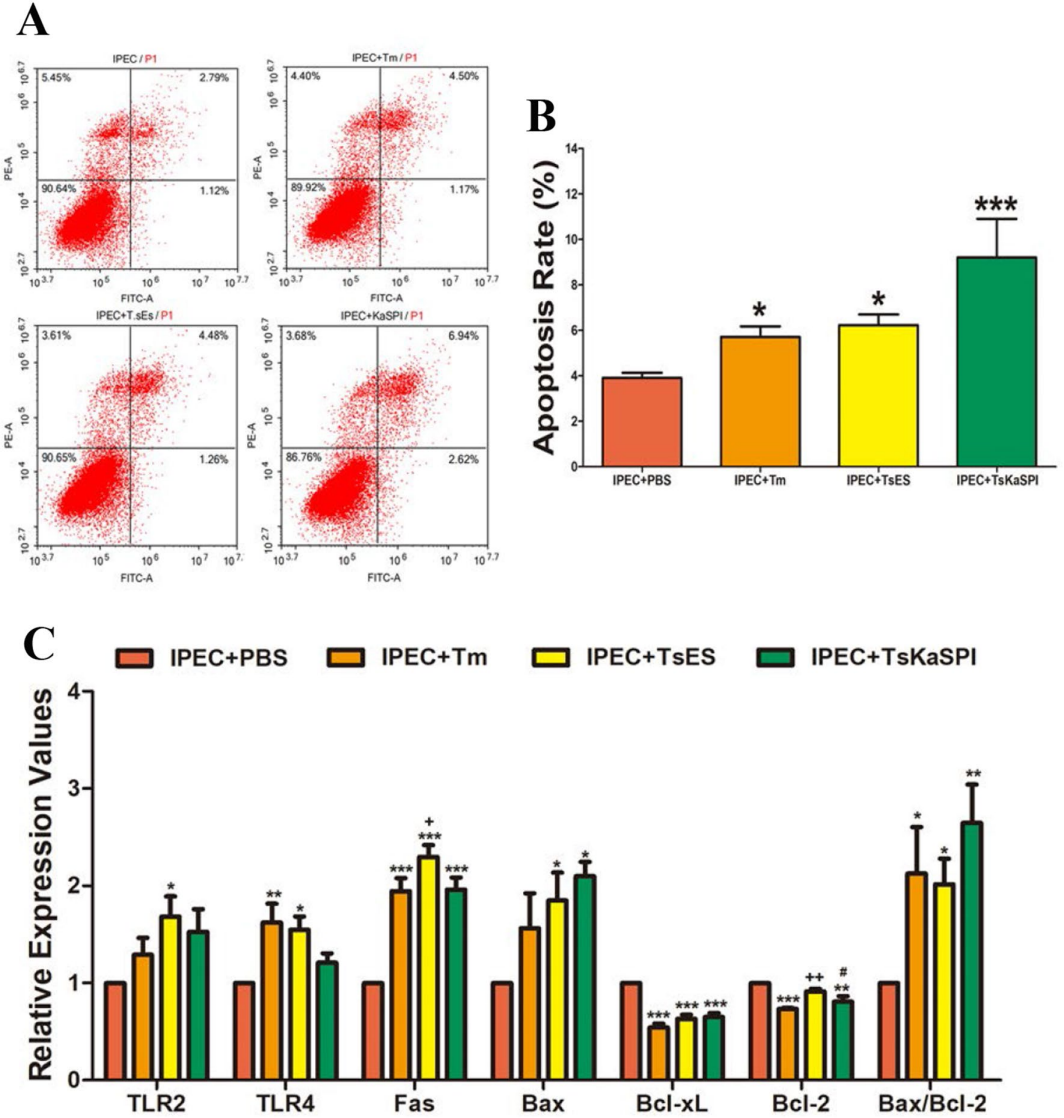
**Tm, TsES, and TsKaSPI affect IPEC apoptosis.** IPEC apoptosis was determined by staining with annexin V and PI followed by flow cytometry (**A**). The percentages of cells with different staining patterns are shown. The results presented are representative of three independent experiments (**B**). qPCR analysis of the relative expression of TLR2, TLR4, proapoptotic genes (Fas, Bax) and antiapoptotic genes (Bcl-xL, Bcl-2) in IPECs (**C**). Assays were performed in triplicate, and the data are presented as the mean ± SD. *P < 0.05, **P < 0.01, ***P < 0.001 compared with the IPEC + PBS group, ^+^*P* < 0.05, ^++^*P* < 0.01, ^+++^*P* < 0.001 compared with the IPEC + Tm group, ^#^*P* < 0.05, ^##^*P* < 0.01, ^###^*P* < 0.001 compared with the IPEC + TsES group.

FCM was used to analyse the apoptosis rates of IPECs. The results showed that Tm, TsES, and TsKaSPI could significantly induce early and late apoptosis in IPECs compared with those in the control group (Figure 8A, B).

qPCR was used to analyse changes in the transcription levels of TLR2, TLR4, proapoptotic (Fas, Bax) genes and antiapoptotic genes (Bcl-xL, Bcl-2). The results are shown in Figure 8C. TsES increased the transcription levels of TLR2 and TLR4 significantly more than those in the control group (*P* < 0.05), while TsKaSPI did not cause significant changes in the transcription levels of TLR2 or TLR4 (*P* > 0.05). Moreover, TsES and TsKaSPI significantly increased the transcription levels of proapoptotic genes and significantly decreased the transcription levels of antiapoptotic genes, and the ratio of Bax/Bcl-2 was significantly higher than that in the control group. Therefore, TsES and TsKaSPI caused apoptosis in IPECs. The qPCR results were consistent with the FCM results.

### TsKaSPI increased the expression of proinflammatory cytokines

**Figure 9:**
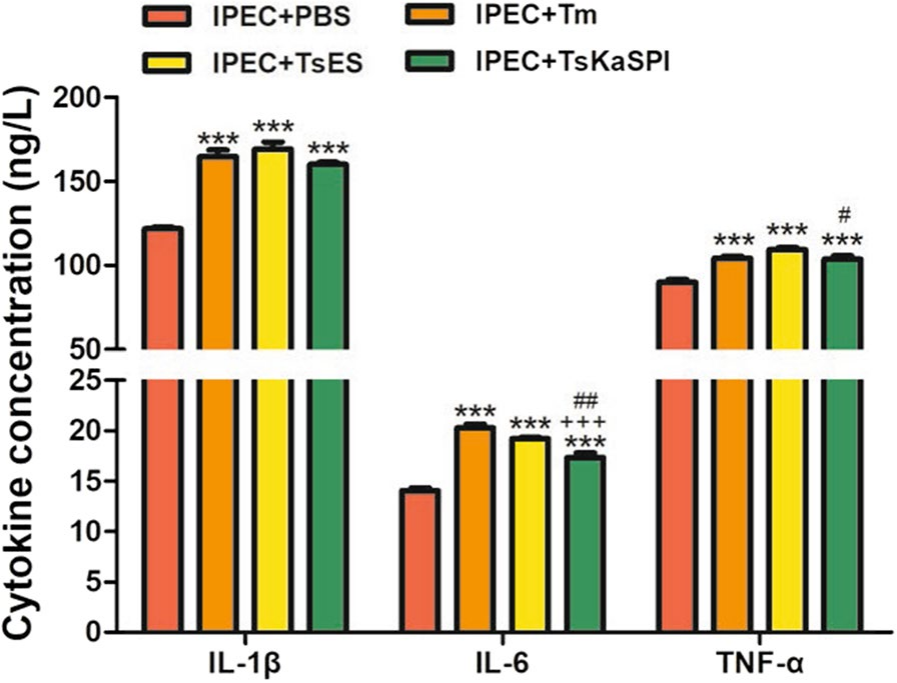
**ELISA analysis of proinflammatory cytokine levels.** The results presented are representative of three independent experiments. The data are presented as the mean ± SD. *P < 0.05, **P < 0.01, ***P < 0.001 compared with the IPEC + PBS group, ^+^*P* < 0.05, ^++^*P* < 0.01, ^+++^*P* < 0.001 compared with the IPEC + Tm group, ^#^*P* < 0.05, ^##^*P* < 0.01, ^###^*P* < 0.001 compared with the IPEC + TsES group.

The effects of Tm, TsES, and TsKaSPI on cytokine production by IPECs were analysed, and the results showed that the production of IL-6, IL-1β and TNF-α was significantly increased compared with that in the control group (Figure 9).

### Inhibition experiments confirmed the interaction between ERS and inflammatory pathways

**Figure 10:**
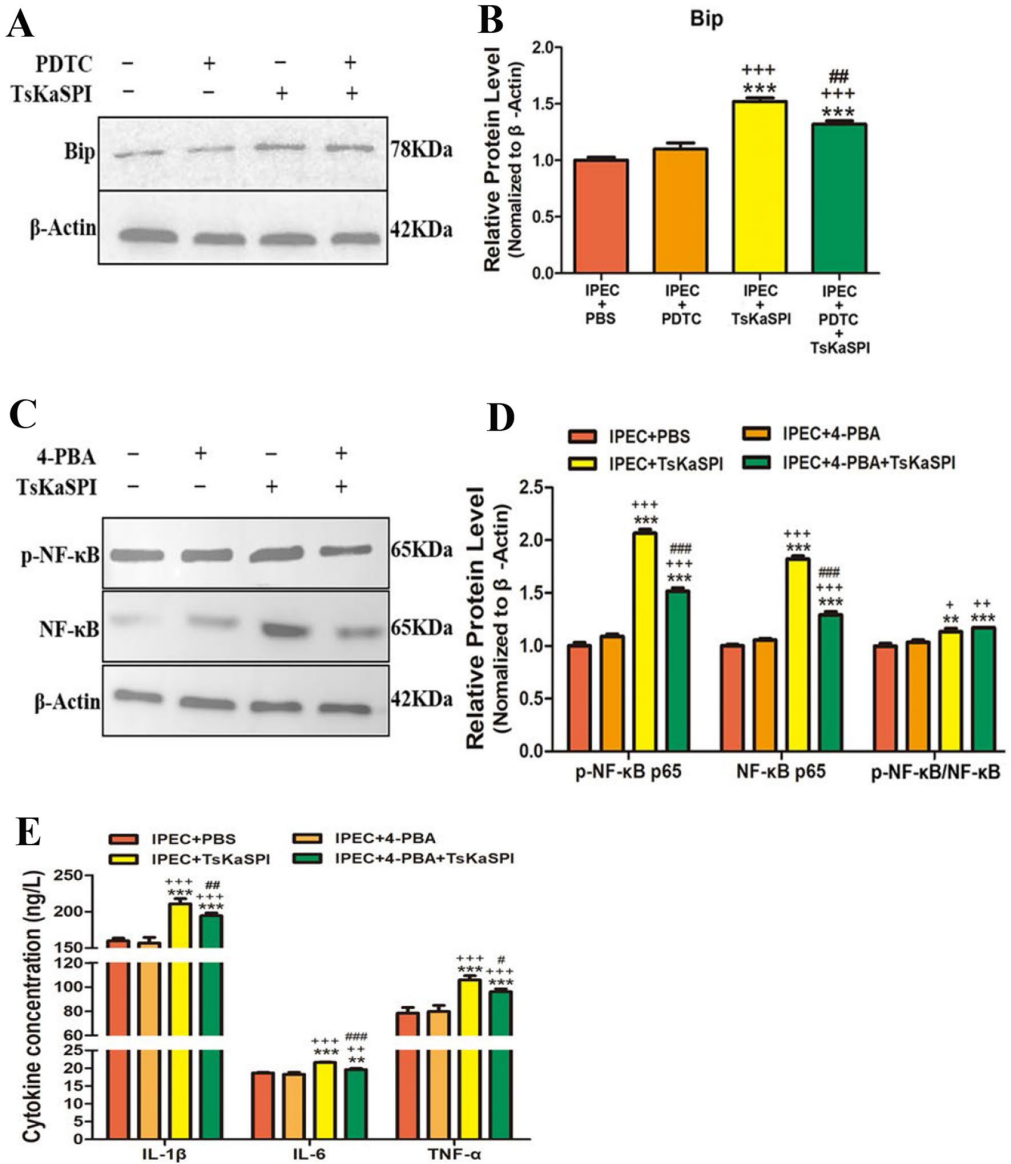
**Inhibition experiment to analyse the relationship between ERS and the NF-κB signalling pathway.** Western blot analysis of the relative expression of Bip after the addition of NF-κB-specific inhibitors (**A**). ImageJ was used to analyse the grey values of the bands, and the results are shown in **B**. Western blot analysis of the relative expression of NF-κB and p-NF-κB after the addition of ERS-specific inhibitors (**C**). ImageJ was used to analyse the grey values of the bands, and the results are shown in **D**. The expression of proinflammatory cytokines was determined by ELISA, and the results are shown in **E**. The data are presented as the mean ± SD. *P < 0.05, **P < 0.01, ***P < 0.001 compared with the IPEC + PBS group, ^+^*P* < 0.05, ^++^*P* < 0.01, ^+++^*P* < 0.001 for IPEC + TsKaSPI, and IPEC + PDTC + TsKaSPI group compared with the IPEC + PDTC group or IPEC + TsKaSPI, and IPEC + 4-PBA + TsKaSPI group compared with the IPEC + 4-PBA group, ^#^*P* < 0.05, ^##^*P* < 0.01, ^###^*P* < 0.001 for IPEC + PDTC + TsKaSPI compared with the IPEC + TsKaSPI group or IPEC + 4-PBA + TsKaSPI compared with the IPEC + TsKaSPI group.

To analyse the association between ERS and the NF-κB signalling pathway, we used 4-PBA (an ERS-selective inhibitor) and PDTC (an NF-κB-specific inhibitor) to perform inhibition experiments. First, Western blotting was used to examine the expression of Bip in the control (IPEC + PBS), IPEC + PDTC, IPEC + TsKaSPI, and IPEC + PDTC + TsKaSPI groups, and the grey values of the bands were analysed by ImageJ software (Figure 10A). The expression of Bip in the IPEC + TsKaSPI and IPEC + PDTC + TsKaSPI groups was significantly higher than that in the control group (*P* < 0.001), and the expression level in the IPEC + TsKaSPI group was significantly higher than that in the IPEC + PDTC + TsKaSPI group (*P* < 0.01) (Figure 10B). These results showed that inhibiting the NF-κB signalling pathway could inhibit TsKaSPI-induced ERS.

Similarly, 4-PBA is a selective ERS inhibitor. We used Western blotting to examine the expression of NF-κB and p-NF-κB in the control (IPEC + PBS), IPEC + 4-PBA, IPEC + TsKaSPI, and IPEC + 4-PBA + TsKaSPI groups, and the grey values of the bands were analysed by ImageJ software (Figure 10C). The protein expression of NF-κB p65 and p-NF-κB p65 in the IPEC + TsKaSPI and IPEC + 4-PBA + TsKaSPI groups was significantly higher than that in the control group (*P* < 0.001), and NF-κB p65 and p-NF-κB p65 expression levels in the IPEC + TsKaSPI groups were significantly higher than those in the IPEC + 4-PBA + TsKaSPI groups (*P* < 0.001). Moreover, the phosphorylation of NF-κB showed the same trend (Figure 10D).

Furthermore, ELISA was used to examine the secretion of IL-6, IL-1β, and TNF-α in the cell supernatants in the groups. The results showed that there was no significant difference between the control group and the IPEC + 4-PBA group. Compared with that in the control group, the secretion of IL-6, IL-1β, and TNF-α in the IPEC + TsKaSPI and IPEC + 4-PBA + TsKaSPI groups was significantly increased. Moreover, the secretion levels in the IPEC + TsKaSPI group were significantly higher than those in the IPEC + 4-PBA + TsKaSPI group (Figure 10E). These results showed that TsKaSPI could activate the NF-κB signalling pathway by inducing ERS, but when ERS was inhibited, TsKaSPI could also activate the NF-κB signalling pathway in other ways.

### TsKaSPI induced ERS in mice

**Figure 11:**
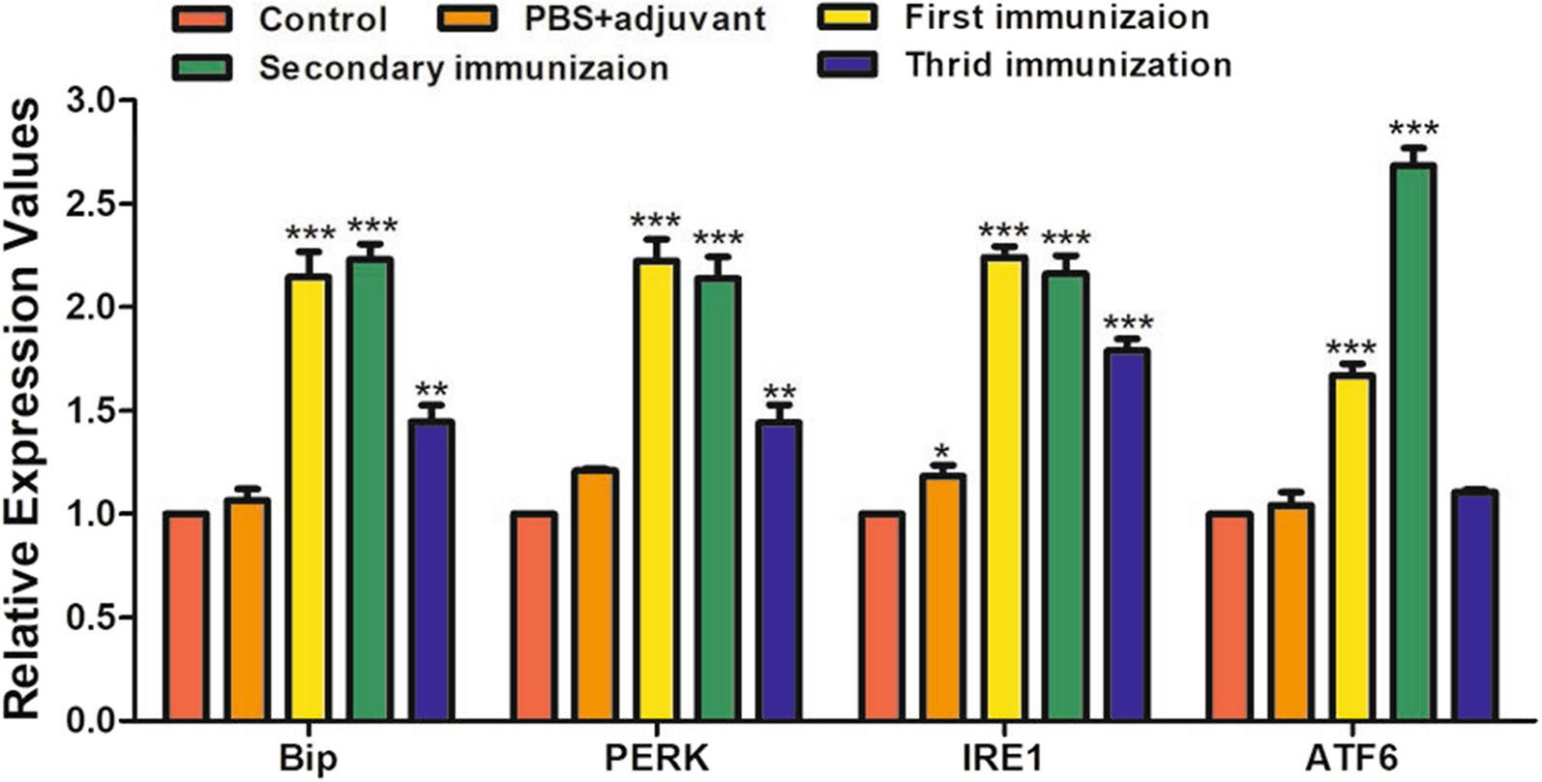
**qPCR analysis of the relative expression of ERS-related proteins in mice immunized with TsKaSPI.** Assays were performed in triplicate, and the data are expressed as the mean ± SD. *P < 0.05, **P < 0.01, ***P < 0.001 compared with the control group.

**Figure 12:**
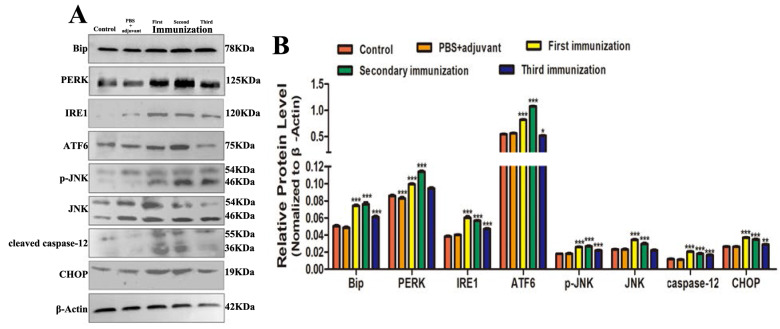
**Western blot analysis of the expression of ERS-related proteins and apoptosis-related proteins in mice that were administered TsKaSPI.** Assays were performed in triplicate, and the data are expressed as the mean ± SD. *P < 0.05, **P < 0.01, ***P < 0.001 compared with the control group.

To evaluate the effect of TsKaSPI on ERS in the jejunum in vivo, BALB/c mice were intraperitoneally administered TsKaSPI three times, and the jejunum was collected. The gene transcription levels of ATF6, Bip, IRE1, and PERK were measured by qPCR, and the protein expression levels of ATF6, Bip, IRE1, PERK, p-JNK, JNK, CHOP, and caspase-12 were measured by Western blotting. Similar to *T. spiralis* infection, TsKaSPI administration significantly increased the expression of ATF6, BIP, IRE1, and PERK (Figure 11). Furthermore, the expression of apoptosis-related proteins was significantly increased by TsKaSPI (Figure 12).

## Discussion

The endoplasmic reticulum (ER) is an important organelle for synthesizing, folding and secreting proteins in eukaryotic cells. However, many factors can cause an imbalance in ER homeostasis, resulting in ER stress (ERS) [35]. As ERS often causes the accumulation of unfolded or misfolded proteins, which induces the unfolded protein response (UPR), the marker molecules involved in the UPR are generally used to indicate the occurrence of ERS. Activating transcription factor 6 (ATF6), protein kinase RNA-like ER kinase (PERK), and inositol requiring enzyme 1 (IRE1) are important molecules of the three UPR signalling pathways [36]. Under non-ERS conditions, immunoglobulin-binding protein (Bip) can bind to these three molecules and maintain the inactive state of the signal transduction factors. When the UPR is induced, Bip dissociates to reduce or stop ERS. Therefore, upregulated Bip expression is often regarded as a marker of the occurrence of ERS [37].

In recent years, many studies have shown that ERS plays a key role in the interaction between the pathogenicity of parasitic infection and the host immune response [38, 39]. Therefore, we conducted a series of experiments to explore whether *Trichinella spiralis* (*T. spiralis*) infection also induced ERS in host intestinal epithelial cells. Gou et al. [40] showed that the expression levels of ERS-related molecules in the jejunum were significantly increased 7 days post-infection (dpi) with *T. spiralis*, while the expression levels of ERS-related molecules in the duodenum were not significantly changed compared with those in the control group. Therefore, we selected jejunum tissue as the research object for this study. First, H&E staining and histological evaluation indicated that *T. spiralis* infection induced intestinal tissue damage (villus loss and ultrastructural damage) at 3 dpi and 7 dpi, and the degree of damage was alleviated to a certain extent at 15 dpi. Moreover, the TUNEL assay showed that the ratio of apoptotic cells was increased at 3, 7, and 15 dpi compared with that in uninfected mice, and the ratio peaked at 7 dpi. The ERS markers ATF6, IRE1, PERK, and Bip increased significantly at 3 dpi and continued until 7 dpi. However, the degree of ERS decreased at 15 dpi. This result may be related to the life cycle of *T. spiralis*. The times points 3 dpi and 7 dpi are the intestinal stage, and 15 dpi is the period when newborn larvae (NBL) migrate in the host’s circulatory system and tissues. Therefore, excessive ERS in intestinal epithelial cells began to gradually alleviate. This finding suggests that ERS occurs in the intestines of mice infected with *T. spiralis* and leads to apoptosis in intestinal epithelial cells.

If ER functions are persistently or severely disrupted, apoptosis pathways such as C/EBP homologue (CHOP), Caspase-12, and/or C-Jun NH2-terminal kinase (JNK) are activated to initiate cell death, which results in an inflammatory response and immune imbalance [41–44]. Therefore, we examined changes in the expression levels of ERS-related apoptosis proteins during *T. spiralis* infection. The results showed that the expression levels of CHOP, cleaved caspase-12 and p-JNK were significantly upregulated in the intestinal stage of infection and gradually decreased in the circulating stage of NBL but were still significantly higher than those in uninfected mice. These results suggest that ERS-mediated apoptosis may play a key role in intestinal invasion and the pathogenesis of *T. spiralis* infection.

During *T. spiralis* infection, the host immune response is mainly regulated by the interaction between excretion/secretion (ES) antigens and the host immune system; therefore, we hypothesized that TsKaSPI, which is one of the important components of ES antigens, plays a key role in the regulation of ERS in the host. Our previous studies confirmed that TsKaSPI was involved in regulating various immune cells in the host and participated in the immune evasion process during *T. spiralis* infection [45, 46]. To further explore the relationship between TsKaSPI and ERS, we conducted in vitro experiments. The results showed that the expression of ERS-related proteins and apoptosis-related proteins increased significantly. Flow cytometry indicated that TsES and TsKaSPI both caused significant increases in the apoptosis rates. The qPCR results showed that TsES and TsKaSPI caused significant increases in the transcription levels of proapoptotic genes and significant decreases in the transcription levels of antiapoptotic genes. In addition, ERS-mediated epithelial apoptosis is associated with the expression of Toll-like receptors (TLRs) [19]. Our results showed that TsES significantly increased the transcription levels of TLR2 and TLR4, while TsKaSPI had no obvious effect on the transcript levels of TLR2 or TLR4.

We further conducted in vivo experiments. BALB/c mice were intraperitoneally injected with TsKaSPI three times, and the jejunum tissues were collected 3 days after each administration. The results showed that ERS-related proteins and apoptosis-related proteins were significantly upregulated after the first, second and third administration. These results suggest that TsKaSPI may play an important role in the process by which *T. spiralis* invades the host intestine and induces ERS in intestinal epithelial cells. Moreover, TsKaSPI also induced apoptosis in intestinal epithelial cells by specifically activating CHOP, caspase-12 and JNK. However, whether other apoptosis pathways, such as death receptor-mediated or mitochondria-targeted apoptosis pathways, are involved in this pathological process remains to be clarified.

During the process by which *T. spiralis* invades the host intestines, mechanical properties and the stimulation of ES antigens can cause extensive intestinal inflammation [4]. Studies have shown that ERS and inflammatory signals in cells share regulators and effectors, and the vicious cycle formed by these two signalling pathways can exacerbate cell dysfunction and cause apoptosis in many cells [46]. As an important multipotent transcription factor, NF-κB plays a key role in regulating the immune response induced by infection [47]. Moreover, our previous experimental results confirmed that TsKaSPI administration could lead to the activation of the NF-κB signalling pathway in the intestinal tissues of mice [44, 45]. Therefore, to determine whether the occurrence of ERS can regulate the immune system through the NF-κB signalling pathway, Western blotting was used to examine the activation of NF-κB in IPECs cultured with Tm, TsES, and TsKaSPI for 24 h. The results showed that Tm, which is an ERS activator, could significantly increase the phosphorylation level of NF-κB. Furthermore, the expression of proinflammatory cytokines also increased significantly. Therefore, these results preliminarily indicated that TsES and TsKaSPI could activate the NF-κB signalling pathway by inducing ERS in IPECs.

However, the role of ERS and inflammation is not unilateral, and inflammatory factors can also activate ERS [48]. Therefore, we further used inhibition experiments to analyse the relationship between ERS and the NF-κB inflammatory signalling pathway. First, 4-PBA was used to limit ERS activation, and the phosphorylation level of NF-κB and the expression of inflammatory cytokines induced by TsKaSPI were significantly decreased. On the other hand, PDTC was used to limit ERS activation, and the degree of ERS was also significantly decreased. Therefore, our results suggested that TsKaSPI-induced ERS in IPECs was accompanied by activation of the NF-κB pathway and that these two pathways were mutually regulated.

In conclusion, both *T. spiralis* and TsKaSPI can induce ERS in host intestinal epithelial cells and cause apoptosis by activating the CHOP, caspase-12 and JNK pathways. Furthermore, the NF-κB signalling pathway and inflammatory factors also play important roles in immune regulation by interacting with ERS. Therefore, TsKaSPI plays an important role during *T. spiralis* invasion in the host.

## Data Availability

The datasets used or analysed during the current study are available from the corresponding author upon reasonable request.

## References

[1] Fu BQ, Li WH, Gai WY, Yao JX, Qu ZG, Xie ZZ, Wang YH, Zhang DL, Blaga R (2013). Detection of anti-Trichinella antibodies in serum of experimentally-infected swine by immunochromatographic strip. Vet Parasitol.

[2] Devleesschauwer B, Praet N, Speybroeck N, Torgerson PR, Haagsma JA, De Smet K, Murrell KD, Pozio E, Dorny P (2015). The low global burden of trichinellosis: evidence and implications. Int J Parasitol.

[3] Hall RL, Lindsay A, Hammond C, Montgomery SP, Wilkins PP, da Silva AJ, McAuliffe I, de Almeida M, Bishop H, Mathison B, Sun B, Largusa R, Jones JL (2012). Outbreak of human trichinellosis in Northern California caused by *Trichinella murrelli*. Am J Trop Med Hyg.

[4] Ding J, Liu X, Bai X, Wang Y, Li J, Wang C, Li S, Liu M, Wang X (2020). *Trichinella spiralis*: inflammation modulator. J Helminthol.

[5] Yi N, Yu P, Wu L, Liu Z, Guan J, Liu C, Liu M, Lu Y (2020). RNAi-mediated silencing of *Trichinella spiralis* serpin-type serine protease inhibitors results in a reduction in larval infectivity. Vet Res.

[6] Yang F, Yang DQ, Song YY, Guo KX, Li YL, Long SR, Jiang P, Cui J, Wang ZQ (2019). In vitro silencing of a serine protease inhibitor suppresses *Trichinella spiralis* invasion, development, and fecundity. Parasitol Res.

[7] Song YY, Zhang Y, Ren HN, Sun GG, Qi X, Yang F, Jiang P, Zhang X, Cui J, Wang ZQ (2018). Characterization of a serine protease inhibitor from *Trichinella spiralis* and its participation in larval invasion of host’s intestinal epithelial cells. Parasites Vectors.

[8] Piekarska J, Szczypka M, Obmińska-Mrukowicz B, Gorczykowski M (2009). Effect of phytohaemagglutinin-P on apoptosis and necrosis in *Trichinella spiralis* infected mice. Vet Parasitol.

[9] Piekarska J, Michalski A, Szczypka M, Obmińska-Mrukowicz B (2009). *Trichinella spiralis*: effect of thymus factor X on apoptosis and necrosis in mice. Exp Parasitol.

[10] Lim YJ, Choi HH, Choi JA, Jeong JA, Cho SN, Lee JH, Park JB, Kim HJ, Song CH (2013). *Mycobacterium kansasii*-induced death of murine macrophages involves endoplasmic reticulum stress responses mediated by reactive oxygen species generation or calpain activation. Apoptosis.

[11] McGuckin MA, Eri RD, Das I, Lourie R, Florin TH (2010). ER stress and the unfolded protein response in intestinal inflammation. Am J Physiol Gastrointest Liver Physiol.

[12] Hosoi T, Ozawa K (2010). Endoplasmic reticulum stress in disease: mechanisms and therapeutic opportunities. Clin Sci.

[13] Duan X, Zhou Y, Teng X, Tang C, Qi Y (2009). Endoplasmic reticulum stress mediated apoptosis is activated in vascular calcification. Biochem Biophys Res Commun.

[14] Yu YR, Deng MJ, Lu WW, Zhang JS, Jia MZ, Huang J, Qi YF (2014). Endoplasmic reticulum stress-mediated apoptosis is activated in intestines of mice with *Trichinella spiralis* infection. Exp Parasitol.

[15] Wang S, Binder P, Fang Q, Wang Z, Xiao W, Liu W, Wang X (2018). Endoplasmic reticulum stress in the heart: insights into mechanisms and drug targets. Br J Pharmacol.

[16] Ghosh R, Colon-Negron K, Papa FR (2019). Endoplasmic reticulum stress, degeneration of pancreatic islet β-cells, and therapeutic modulation of the unfolded protein response in diabetes. Mol Metab.

[17] Schwarz DS, Blower MD (2016). The endoplasmic reticulum: structure, function and response to cellular signaling. Cell Mol Life Sci.

[18] Roberson EC, Tully JE, Guala AS, Reiss JN, Godburn KE, Pociask DA, Alcorn JF, Riches DW, Dienz O, Janssen-Heininger YM, Anathy V (2012). Influenza induces endoplasmic reticulum stress, caspase-12-dependent apoptosis, and c-Jun N-terminal kinase-mediated transforming growth factor-β release in lung epithelial cells. Am J Respir Cell Mol Biol.

[19] Hausmann M (2010). How bacteria-induced apoptosis of intestinal epithelial cells contributes to mucosal inflammation. Int J Inflam.

[20] Morada M, Pendyala L, Wu G, Merali S, Yarlett N (2013). *Cryptosporidium parvum* induces an endoplasmic stress response in intestinal adenocarcinoma HCT-8 cell line. J Biol Chem.

[21] Dias-Teixeira KL, Calegari-Silva TC, dos Santos GR, Vitorino Dos Santos J, Lima C, Medina JM, Aktas BH, Lopes UG (2016). The integrated endoplasmic reticulum stress response in *Leishmania amazonensis* macrophage infection: the role of X-box binding protein 1 transcription factor. FASEB J.

[22] Yu YR, Ni XQ, Huang J, Zhu YH, Qi YF (2016). Taurine drinking ameliorates hepatic granuloma and fibrosis in mice infected with *Schistosoma japonicum*. Int J Parasitol Drugs Drug Resist.

[23] Wang T, Zhou J, Gan X, Wang H, Ding X, Chen L, Wang Y, Du J, Shen J, Yu L (2014). *Toxoplasma gondii* induce apoptosis of neural stem cells via endoplasmic reticulum stress pathway. Parasitology.

[24] Gu Y, Wei J, Yang J, Huang J, Yang X, Zhu X (2013). Protective immunity against *Trichinella spiralis* infection induced by a multi-epitope vaccine in a murine model. PLoS One.

[25] Yang X, Yang Y, Wang Y, Zhan B, Gu Y, Cheng Y, Zhu X (2014). Excretory/secretory products from *Trichinella spiralis* adult worms ameliorate DSS-induced colitis in mice. PLoS One.

[26] Ilic N, Worthington JJ, Gruden-Movsesijan A, Travis MA, Sofronic-Milosavljevic L, Grencis RK (2011). *Trichinella spiralis* antigens prime mixed Th1/Th2 response but do not induce de novo generation of Foxp3+ T cells in vitro. Parasite Immunol.

[27] Dea-Ayuela MA, Rama-Iñiguez S, Bolas-Fernández F (2006). Vaccination of mice against intestinal *Trichinella spiralis* infections by oral administration of antigens microencapsulated in methacrilic acid copolymers. Vaccine.

[28] Zhang Z, Mao Y, Li D, Zhang Y, Li W, Jia H, Zheng J, Li L, Lu Y (2016). High-level expression and characterization of two serine protease inhibitors from *Trichinella spiralis*. Vet Parasitol.

[29] Yang GY, Yu J, Su JH, Jiao LG, Liu X, Zhu YH (2017). Oral administration of *Lactobacillus rhamnosus* GG ameliorates *Salmonella Infantis*-induced inflammation in a pig model via activation of the IL-22BP/IL-22/STAT3 pathway. Front Cell Infect Microbiol.

[30] Zhou D, Zhu YH, Zhang W, Wang ML, Fan WY, Song D, Yang GY, Jensen BB, Wang JF (2015). Oral administration of a select mixture of *Bacillus* probiotics generates Tr1 cells in weaned F4ab/acR pigs challenged with an F4+ ETEC/VTEC/EPEC strain. Vet Res.

[31] Livak KJ, Schmittgen TD (2001). Analysis of relative gene expression data using real-time quantitative PCR and the 2(−Delta Delta C(T)) method. Methods.

[32] Fernández-Blanco JA, Hollenberg MD, Martínez V, Vergara P (2013). PAR-2-mediated control of barrier function and motility differs between early and late phases of postinfectious gut dysfunction in the rat. Am J Physiol Gastrointest Liver Physiol.

[33] Muñoz-Carrillo JL, Muñoz-Escobedo JJ, Maldonado-Tapia CH, Chávez-Ruvalcaba F, Moreno-García MA (2017). Excretory/secretory products from *Trichinella spiralis* adult worms ameliorate DSS-induced colitis in mice. Parasite Immunol.

[34] Zhou J, Gan X, Wang Y, Zhang X, Ding X, Chen L, Du J, Luo Q, Wang T, Shen J, Yu L (2015). *Toxoplasma gondii* prevalent in China induce weaker apoptosis of neural stem cells C17.2 via endoplasmic reticulum stress (ERS) signaling pathways. Parasites Vectors.

[35] Zhang C (2017). Roles of Grp78 in female mammalian reproduction. Adv Anat Embryol Cell Biol.

[36] Morada M, Pendyala L, Wu G, Merali S, Yarlett N (2013). *Cryptosporidium parvum* induces an endoplasmic stress response in the intestinal adenocarcinoma HCT-8 cell line. J Biol Chem.

[37] Lu Y, Yang Y, Yang S, Xia Q (2020). Immunomodulatory action of excretory-secretory products of *Angiostrongylus cantonensis* in a mouse tumour model. Parasitol Res.

[38] Chen KY, Chen YJ, Cheng CJ, Jhan KY, Wang LC (2020). Excretory/secretory products of *Angiostrongylus cantonensis* fifth-stage larvae induce endoplasmic reticulum stress via the Sonic hedgehog pathway in mouse astrocytes. Parasites Vectors.

[39] Weingartner M, Stücheli S, Jebbawi F, Gottstein B, Beldi G, Lundström-Stadelmann B, Wang J, Odermatt A (2022). Albendazole reduces hepatic inflammation and endoplasmic reticulum-stress in a mouse model of chronic *Echinococcus multilocularis* infection. PLoS Negl Trop Dis.

[40] Gou Q (2014) Study on the alternation of IRE1 pathway in intestinal gene expressions and cytokines in intestine of the mouse infected with the parasite *Trichinella spiralis*. Jilin Agricultural University

[41] Nakagawa T, Zhu H, Morishima N, Li E, Xu J, Yankner BA, Yuan J (2000). Caspase-12 mediates endoplasmic-reticulum-specifific apoptosis and cytotoxicity by amyloid-beta. Nature.

[42] Urano F, Wang X, Bertolotti A, Zhang Y, Chung P, Harding HP, Ron D (2000). Coupling of stress in the ER to activation of JNK protein kinases by transmembrane protein kinase IRE1. Science.

[43] Xu J, Wu L, Yu P, Sun Y, Lu Y (2020). Effect of *T. spiralis* serine protease inhibitors on TNBS-induced experimental colitis mediated by macrophages. Sci Rep.

[44] Xu J, Wu L, Yu P, Liu M, Lu Y (2018). Effect of two recombinant *Trichinella spiralis* serine protease inhibitors on TNBS-induced experimental colitis of mice. Clin Exp Immunol.

[45] Li W, Cao T, Luo C, Cai J, Zhou X, Xiao X, Liu S (2020). Crosstalk between ER stress, NLRP3 inflammasome, and inflammation. Appl Microbiol Biotechnol.

[46] Lawrence T (2009). The nuclear factor NF-kappaB pathway in inflammation. Cold Spring Harb Perspect Biol.

[47] Hayden MS, Ghosh S (2011). NF-κB in immunobiology. Cell Res.

[48] Shkoda A, Rui PA, Daniel H, Kim SC, Rogler G, Sartor RB, Haller D (2007). Interleukin-10 blocked endoplasmic reticulum stress in intestinal epithelial cells: impact on chronic inflammation. Gastroenterology.

